# Preparation of Monodisperse Silica Mesoporous Microspheres with Narrow Pore Size Distribution

**DOI:** 10.3390/polym16121724

**Published:** 2024-06-17

**Authors:** Jiaqi Shan, Jia Liu, Jiahui Zhu, Lifei Chen, Ting Xu, Xingfa Ren, Xingzhong Guo

**Affiliations:** 1 State Key Laboratory of Silicon and Advanced Semiconductor Materials, School of Materials Science and Engineering, Zhejiang University, Hangzhou 310027, China; 21626008@zju.edu.cn; 2 ZJU—Hangzhou Global Scientific and Technological Innovation Center, Hangzhou 311200, China; 3 Zhejiang X–Way Nano Technology Co., Ltd., Hangzhou 311200, China; liujia-xway@outlook.com (J.L.); zhujiahui950323@outlook.com (J.Z.); q463925892@outlook.com (L.C.); 4 Zhejiang Welch Materials Technology Co., Ltd., Jinhua 321016, China; tingxu1@welchmat.com (T.X.); xingfaren@welchmat.com (X.R.)

**Keywords:** monodisperse microspheres, emulsion method, post–treatment, pore structure

## Abstract

The purpose of this study is to prepare monodisperse silica mesoporous microspheres with narrow pore size distribution to promote their application in the field of liquid chromatography. An improved emulsion method was used to prepare silica mesoporous microspheres, and the rotary evaporation temperature, emulsification speed, dosage of porogen DMF, and dosage of the catalyst NH_3_·H_2_O were optimized. Subsequently, these microspheres were respectively treated by alkali–heating, calcination, and sieving. The D_50_ (particle size at the cumulative particle size distribution percentage of 50%) of as–prepared silica mesoporous microspheres is 26.3 μm, and the D_90_/D_10_ (the ratio of particle size at a cumulative particle size distribution percentage of 90% to a cumulative particle size distribution percentage of 10%) is 1.94. The resultant silica mesoporous microspheres have distinctive pore structures, with a pore volume of more than 1.0 cm^3^/g, an average pore size of 11.35 nm, and a median pore size of 13.4 nm. The silica mesoporous microspheres with a large particle size, uniform particle size distribution, large average pore size and pore volume, and narrow mesopore size distribution can basically meet the requirements of preparative liquid chromatographic columns.

## 1. Introduction

Porous materials are a class of materials with distinctive pore structures, in which pores serve as the functional phase. Porous materials can be divided into macroporous materials (pore size of >50 nm), mesoporous materials (pore size of 2–50 nm), and microporous materials (pore size of <2 nm) based on their pore size [1]. Silica mesoporous microspheres, which can be prepared by spray drying, polymerization–induced colloid aggregation (PICA), emulsion, template, sol–gel, and other methods [2,3], have physical stability, controllable particle size, no obvious cytotoxicity, good biocompatible performance, huge specific surface area and pore volume, excellent loading performance, uniform pore size, ordered arrangement, and controllable size and silanol groups [4]. Thanks to their unique properties, silica mesoporous microspheres exhibit promising applications in various fields, such as catalysis and drug delivery [5,6,7,8], liquid chromatography [9,10,11,12], heavy metal adsorption [13,14,15], functional coatings [16,17], and so on. Notably, in liquid chromatography, the stationary phase is primarily composed of silica microspheres, accounting for over 80%.

Liquid chromatography columns can be divided into analytical columns and preparative columns based on their intended use [18,19,20]. Analytical columns are used for the detection and analysis of substances and impurities, demanding high precision and sensitivity, with a minimal injection volume of 10 mg [21,22]. Preparative columns are used for the purification and separation of substances, demanding high separation efficiency and yield, with a large injection volume (>0.1 g) [23,24,25]. Since the injection volume of preparative columns is 10 times or even 100 times that of analytical columns, the amount of silica mesoporous microspheres required for preparative chromatography is also much larger than that for analytical chromatography. Despite the tremendous market demand, there are only limited reports on the synthesis of silica microspheres for preparative liquid chromatographic columns and the corresponding methods. This could be attributed to the fact that most of the silica mesoporous microspheres and their preparation methods fall short of meeting the specifications for preparative chromatography columns. Firstly, liquid chromatography columns are commonly utilized for separation and purification purposes. The stationary phase, which consists of silica mesoporous microspheres, must possess extremely high purity. Secondly, preparative chromatography columns are typically of large dimensions. To uniformly fill these columns, dynamic axial compression [26] is necessary. However, this method is only suitable for particles with diameters exceeding 20 μm. Hence, the silica mesoporous microspheres used in preparative chromatography columns generally have diameters greater than 20 μm. Finally, preparative chromatographic columns demand higher separation efficiency. To facilitate the rapid passage of a large volume of mobile phase, the silica microspheres used must possess uniform particle size distribution, large pore volume, interconnected mesopores, and uniform pore size distribution. The spray drying method can rapidly synthesize high–purity silica microspheres with a diameter greater than 20 μm, but the particle size distribution of the microspheres synthesized by this method is wide and the pore structure is uncontrollable [27,28]. The PICA and sol–gel methods synthesize silica microspheres with intermolecular force as the driving force. The synthesized microspheres have uniform particle size distribution and controllable pore structure, but their diameter is often less than 10 μm [29,30,31,32]. The template method and emulsion method can prepare silica microspheres with a particle size greater than 20 μm and a uniform mesoporous structure, but a large amount of template and surfactant are often used in the preparation process, which seriously affects the purity of the microspheres [33,34,35,36].

In this paper, an improved emulsion method without surfactant and template was used to synthesize silica microspheres with a large particle size, and the silica mesoporous microspheres were achieved by three post–treatment processes of alkali–heating, calcination, and sieving. *N*,*N*–dimethylformamide (DMF), a non–protic polar solvent, was used as a porogen as well as a surfactant to eliminating macromolecular polymer templates and surfactants. The pore size and volume of microspheres were enhanced through alkali–heating. Sieving ensured a narrower particle size distribution for the microspheres. Then, the particle size, particle size distribution, pore volume, and pore size distribution of the silica mesoporous microspheres were investigated in detail. The as–prepared silica mesoporous microspheres can meet the requirements of preparative liquid chromatography columns.

## 2. Materials and Methods

Materials: Tetraethoxysilane (TEOS, Shanghai Aladdin Biochemical Technology Co., Ltd., Shanghai, China, 99%, GC), ultra–pure water (H_2_O, Shanghai Yuanye Bio–Technology Co., Ltd., Shanghai, China), N,N–dimethylformamide (DMF, Sinopharm Chemical Reagent Co., Ltd., Shanghai, China, 99.5%, AR), ethanol (EtOH, Sinopharm Chemical Reagent Co., Ltd., Shanghai, China, 99.7%, AR), isopropanol (IPA, Sinopharm Chemical Reagent Co., Ltd., Shanghai, China, 99.7%, AR), aqueous ammonia (NH_3_·H_2_O, Shanghai Aladdin Biochemical Technology Co., Ltd., Shanghai, China, 25–28%, GR), and hydrochloric acid (HCl, Sinopharm Chemical Reagent Co., Ltd., Shanghai, China, 36–38%, AR) were used as obtained.

Synthesis process of silica microspheres: The HCl concentration was diluted to 0.1 mol/L with H_2_O. The TEOS was dispersed in EtOH, and the diluted HCl solution was dropped at a rate of 0.5 mL/min and then stirred for 1.5 h. The resultant solution was poured into a rotary evaporator and polycondensated at 60 °C and 250 hpa for 1 h to obtain silicone oil. The silicone oil and DMF were mixed evenly, poured into 40 wt% IPA/H_2_O solution, and emulsified at 800 rpm for 10 min to obtain emulsion. The NH_3_·H_2_O and H_2_O were mixed and added to the emulsion and then emulsified at 800 rpm for 20 min. After emulsification, the emulsion was aged for 24 h. After aging, the resultant sediment was washed with ethanol 3 times and dried at 60 °C to obtain the silica microspheres. The volume ratio of TEOS, HCl (0.1 mol/L), ethanol, DMF, IPA/H_2_O (40 wt%), NH_3_·H_2_O, and H_2_O used in this process was 1:0.12:0.6:0.048:6:0.08:0.2.

Post–treatment process of silica microspheres: The NH_3_·H_2_O was diluted to 0.14 mol/L with H_2_O, and the microspheres and diluted NH_3_·H_2_O with a mass ratio of 1:10 were mixed. Subsequently, the microspheres were subjected to alkali–heating reaction at 70 °C for 8 h. After the reaction, the microspheres were washed with H_2_O and EtOH and dried at 60 °C. After drying, the microspheres were put into a muffle furnace and calcined at 650 °C for 5 h to obtain silica mesoporous microspheres. The silica mesoporous microspheres were put into an ultrasonic–vibration sieve, and the monodisperse silica mesoporous microspheres were obtained by sieving with 40 and 20 μm sieves.

Characterization of silica microspheres: The microstructures of the microspheres were observed by a scanning electron microscope (SEM, Hitachi Corp, Tokyo, Japan, SU8010). The pore structures of the microspheres were characterized by a fully automatic surface area and porosity analyzer (BET, Micromeritics Instrument Corp., Norcross, GA, USA, ASAP2460). The particle size distributions of the microspheres were measured by a Mastersizer (PSD, Beckman Coulter Inc., Breya, CA, USA, LS200).

## 3. Results

**Figure 1:**
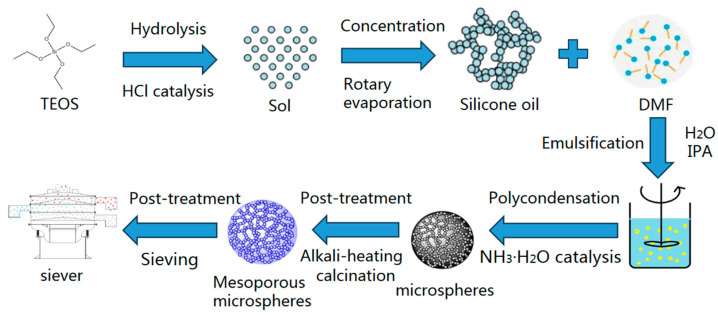
A schematic representation of the preparation process for monodisperse silica mesoporous microspheres.

The preparation process of monodisperse silica mesoporous microspheres is illustrated in Figure 1. The synthesis of silica microspheres mainly includes hydrolysis, concentration, emulsification, and polycondensation. Monodisperse silica mesoporous microspheres are obtained through the post–treatment of silica microspheres, such as alkali–heating, calcination, and sieving. The study systematically optimizes the synthesis and post–treatment processes of silica microspheres. The formula and process characteristics of the prepared samples are detailed in Table 1.

### 3.1. Synthesis of the Silica Microspheres

The preparation process of silica mesoporous microspheres can be divided into the synthesis process and the post–treatment process. In the synthesis process, the silicon precursor undergoes hydrolysis through stirring to form a sol, which then undergoes polymerization through rotary evaporation to form a highly polymerized silicone oil. The silicone oil and the porogen DMF are dispersed in an alcohol–water mixture and form an oil–in–water emulsion under shear force, followed by the addition of catalyst NH_3_·H_2_O to initiate the emulsion polymerization reaction, leading to the formation of silica microspheres. The synthesized silica microspheres also need to undergo post–treatment processes, including alkali–heating, calcination, and sieving. To achieve silica mesoporous microspheres with excellent morphology and pore structure, the effects of synthesis process parameters, such as rotary evaporation temperature, emulsification speed, porogen DMF, catalyst NH_3_·H_2_O, and post–treatment processes, on the microspheres’ morphology and pore structure are investigated in detail.

**Figure 2:**
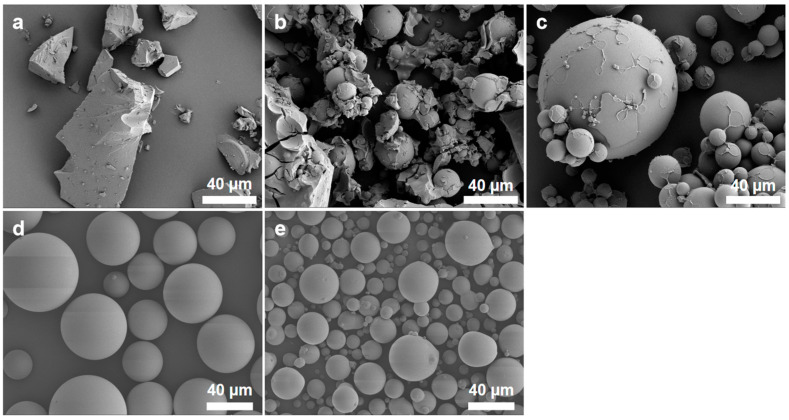
SEM images of the samples synthesized under various rotary evaporation temperatures. (**a**) 50 °C; (**b**) 53 °C; (**c**) 57 °C; (**d**) 60 °C; (**e**) 65 °C.

The variation in rotary evaporation temperature during the synthesis process significantly impacts the morphology of the final product. Figure 2 depicts the microstructure of the samples synthesized under various rotary evaporation temperatures. When the rotary evaporation temperature is set too low for synthesis, the sample exists in the form of an irregular block. As the rotary evaporation temperature rises, the microspheres gradually emerge in the synthesized samples. Further elevation of the rotary evaporation temperature results in the disappearance of irregular blocks from the sample, but there is cohesion between the microspheres with a significant disparity in particle size. By the time the rotary evaporation temperature reaches 60 °C, the sample presents as scattered microspheres, each with an intact spherical shape and free of small particles on its surface. However, when the rotary evaporation temperature exceeds a suitable range, the sphericity of the microspheres notably diminishes, and small particles adhere to their surfaces.

**Figure 3:**
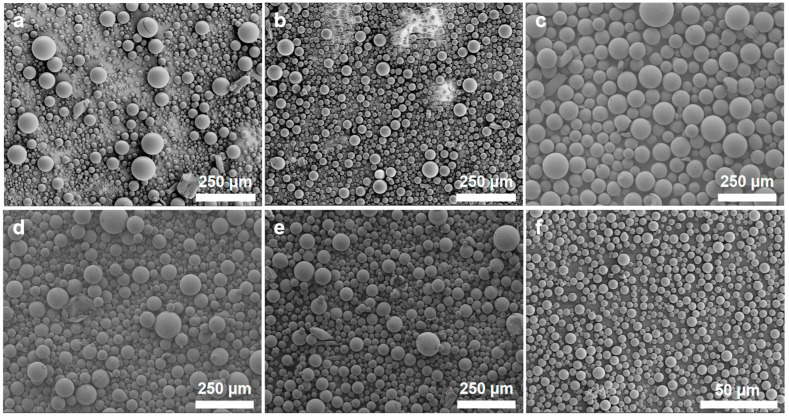
SEM images of the silica microspheres synthesized under various emulsification speeds. (**a**) 600 rpm; (**b**) 700 rpm; (**c**) 800 rpm; (**d**) 1000 rpm; (**e**) 1200 rpm; (**f**) 8000 rpm.

The emulsification speed is crucial for determining the size of silica microspheres and also influences their particle size distribution. Figure 3 depicts the microstructure of the silica microspheres synthesized under various emulsification speeds. When the emulsification speed is increased from 800 to 8000 rpm, the particle size of the synthesized silica microspheres significantly decreases. Although the change in emulsification speed within a small range (600–1200 rpm) does not have a significant impact on the size of microspheres, it does have a certain impact on the particle size distribution of the microspheres. Specifically, the synthesized silica microspheres exhibit a more concentrated particle size distribution under SEM when the emulsification speed is 800 rpm.

**Figure 4:**
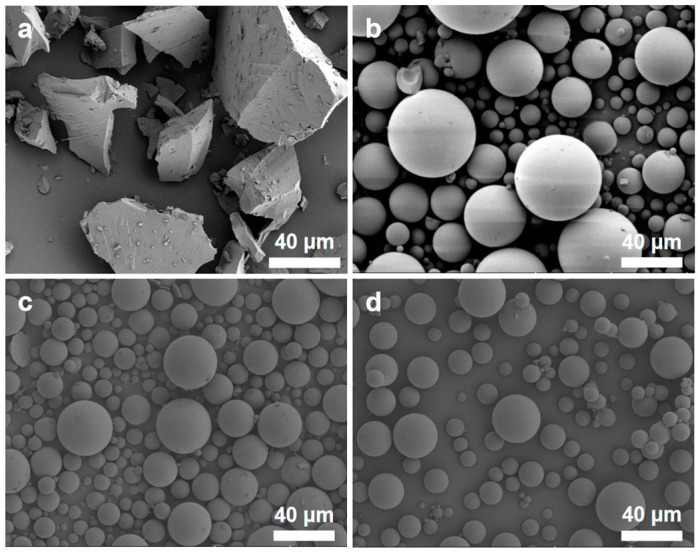
SEM images of the samples synthesized with various dosages of DMF. (**a**) 7.20 mL; (**b**) 9.60 mL; (**c**) 12.0 mL; (**d**) 14.4 mL.

**Figure 5:**
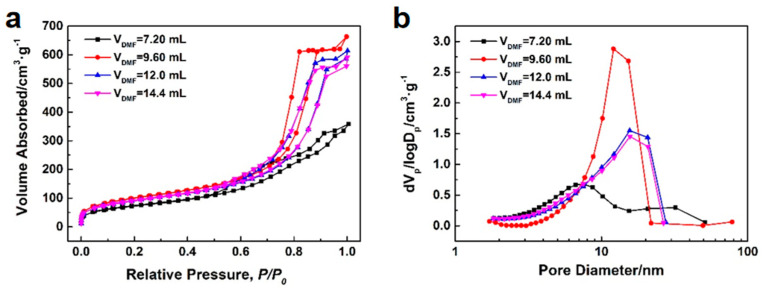
N_2_ adsorption–desorption isotherms (**a**) and BJH mesopore size distributions (**b**) of the samples synthesized with various DMF contents.

DMF acts as both a dispersant and a porogen during the synthesis process, and its content significantly influences the morphology and pore structure of the synthesized samples. Figure 4 depicts the microstructure of the samples synthesized with various DMF content. When the addition of DMF is insufficient, the microstructure of the sample appears as irregular blocks. However, once the DMF content is increased to a certain level, silica microspheres are obtained. Further increasing the DMF content does not lead to significant changes in the morphology of the microspheres. Figure 5a,b show the N_2_ adsorption–desorption isotherms and BJH mesopore size distributions of the samples synthesized with various DMF contents. The pore structure characteristics of the samples are listed in Table 2. Based on the classification of IUPAC, all N_2_ adsorption–desorption isotherms in Figure 5a exhibit type IV, which is characteristic of mesoporous materials. All type IV isotherms exhibit hysteresis loops, of which there are four types: H1, H2, H3, and H4. The shape of hysteresis loops is closely related to the shape of mesopores. When the DMF content is 7.20 mL, the hysteresis loop of the sample belongs to type H3, which indicates that there are uneven slit–like pores in the mesoporous materials. With the increase of DMF content to 9.60 mL, the hysteresis loop of the sample changes to type H1, which implies that there exist cylindrical slender channels with uniform pore sizes. As the DMF content further increases to 12.0 or 14.4 mL, the hysteresis loops of the samples become type H2, which suggests that bottle–like pores mostly exist in mesoporous materials. In addition to variations in pore shape, the DMF content significantly impacts the pore volume, pore size, and pore size distribution of the samples. As depicted in Figure 5b and Table 2, with an increase in DMF content, the median pore size of the samples gradually increases. When the DMF content reaches 9.60 mL, the BJH pore size distribution curve of the sample exhibits a steeper peak, with a smaller difference between the average and median pore sizes, signifying a more concentrated and narrow pore size distribution. Moreover, the sample demonstrates enhanced pore volume and average pore diameter, reflecting superior pore structure characteristics.

**Figure 6:**
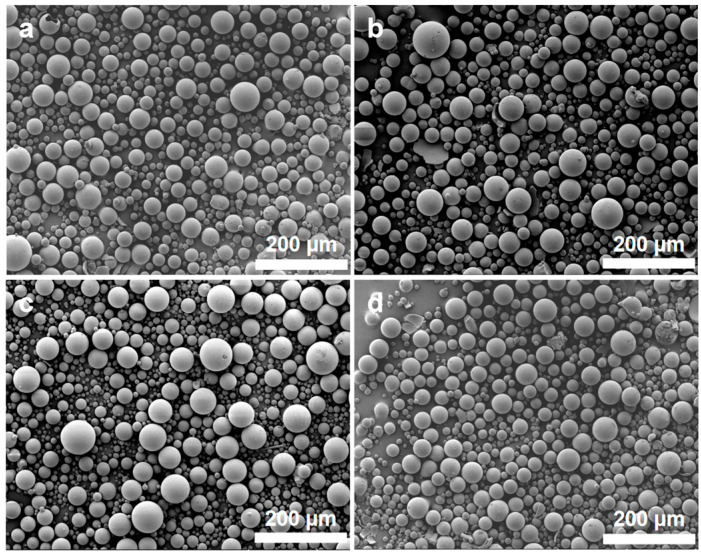
SEM images of the silica microspheres synthesized with various NH_3_·H_2_O contents. (**a**) 8 mL; (**b**) 12 mL; (**c**) 16 mL; (**d**) 20 mL.

**Figure 7:**
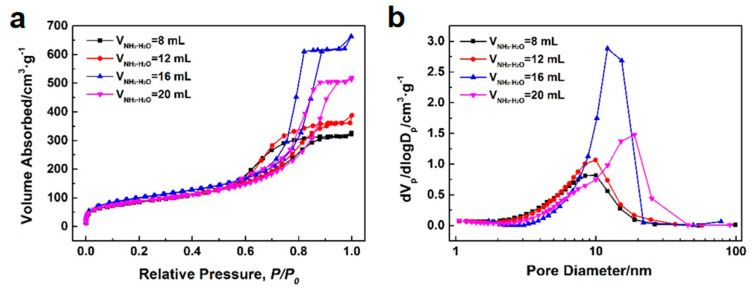
N_2_ adsorption–desorption isotherms (**a**) and BJH mesopore size distributions (**b**) of the silica microspheres synthesized with various NH_3_·H_2_O contents.

Figure 6 depicts the microstructure of the silica microspheres synthesized with various NH_3_·H_2_O contents. As the NH_3_·H_2_O content increases, there is no significant change in the morphology of silica microspheres, except for some damaged spherical shells in the sample synthesized with 8 mL of NH_3_·H_2_O. The NH_3_·H_2_O content has a more pronounced impact on the pore structure of mesoporous silica microspheres. Figure 7a,b show the N_2_ adsorption–desorption isotherms and BJH mesopore size distributions of the silica microspheres synthesized with various NH_3_·H_2_O contents. The pore structure characteristics of the silica microspheres are listed in Table 3. All N_2_ adsorption–desorption isotherms in Figure 7a exhibit type IV, which is characteristic of mesoporous materials. All hysteresis loops in Figure 7a exhibit type H1, which indicates that cylindrical slender channels with uniform pore size exist in the microspheres. As depicted in Figure 7b and Table 3, with an increase in NH_3_·H_2_O content, the median pore size of the samples gradually increases. When the NH_3_·H_2_O content reaches 16 mL, the BJH pore size distribution curve of the sample displays a steeper peak, with a smaller difference between the average and median pore sizes, signifying a more concentrated and narrow pore size distribution. Moreover, the sample demonstrates enhanced pore volume and average pore diameter, showing superior pore structure characteristics.

### 3.2. Post–Treatment of the Silica Microspheres

**Figure 8:**
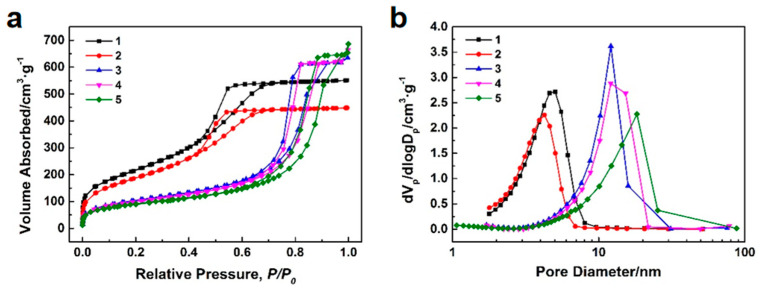
N_2_ adsorption–desorption isotherms (**a**) and BJH mesopore size distributions (**b**) of the silica microsphere samples 1–5 with different post–treatments.

The objective of alkali–heating is to enlarge the pore size and pore volume of the silica microspheres. The aim of calcination is to enhance the strength of the microspheres and remove the residual organic compound, although the calcination process can also impact the pore structure of the microspheres. Hence, alkali–heating and calcination are considered together. To validate the impact of these processes on the pore structure of the microspheres, various post–treatments were applied during the preparation of samples 1–5, as outlined in Table 4. Figure 8a,b show the N_2_ adsorption–desorption isotherms and BJH mesopore size distributions of the silica microsphere samples 1–5 with different post–treatments. The pore structure characteristics of the silica microsphere samples 1–5 with different post–treatments are listed in Table 5. All N_2_ adsorption–desorption isotherms in Figure 8a exhibit type IV, indicating that the microspheres are mesoporous materials. All hysteresis loops in Figure 8a belongs to type H1, which confirms the existence of cylindrical slender channels with uniform pore size. Upon comparing the BJH mesopore size distribution curves and pore structure characteristics of samples 1 and 2, it is evident that the specific surface area, pore volume, and pore size of the silica microspheres have slightly decreased after calcination. Upon comparing the BJH mesopore size distribution curves and pore structure characteristics of samples 2 and 4, it is observed that the specific surface area of the silica microspheres significantly decreases, the pore size significantly increases, and the pore volume slightly increases after alkali–heating, confirming the effect of pore expansion. Upon comparing the BJH mesopore size distribution curves and pore structure characteristics of samples 3–5, it is observed that as the concentration of NH_3_·H_2_O in the alkaline solution used for the alkali–heating reaction increases, the median pore size of the silica mesoporous microspheres gradually increases. However, an excessively high concentration of NH_3_·H_2_O does not significantly increase the average pore size of the silica mesoporous microspheres, leading to non–uniformity in the mesopore size distribution. Among all the samples, sample 4 exhibits a larger pore volume, a relatively larger average pore size, and a narrower pore size distribution.

**Figure 9:**
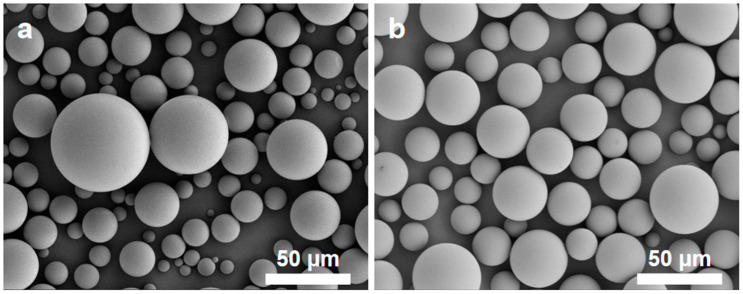
SEM images of the silica microspheres before (**a**) and after (**b**) sieving.

**Figure 10:**
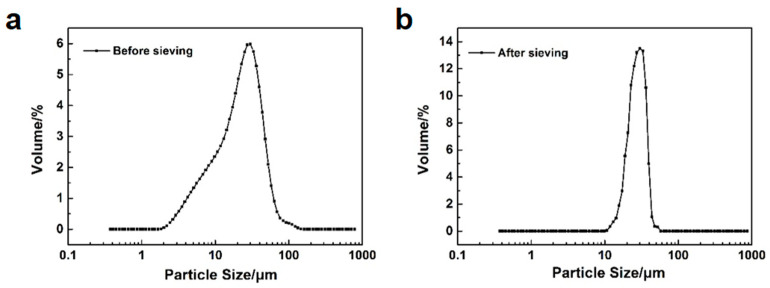
Particle size distributions of the silica microspheres before (**a**) and after (**b**) sieving.

Given the constraints of our laboratory equipment, the particle size distribution of the synthesized silica microspheres remains broad, necessitating sieving. By sieving, the microspheres with either overly large or overly small particle sizes can be removed, achieving monodisperse silica microspheres with a more uniform particle size distribution. Figure 9 depicts the microstructure of the silica microspheres before and after sieving. After sieving, the silica microspheres retain their spherical integrity, showing no signs of damage, and exhibit a more uniform particle size distribution. Figure 10 presents the particle size distribution curves for the silica microspheres before and after sieving, and Table 6 lists the corresponding particle size distribution data. After sieving, the D_90_/D_10_ value of the silica microspheres drops from 6.99 to 1.94, further confirming that the silica microspheres after sieving possess a narrower particle size distribution.

### 3.3. Chromatographic Performance of Silica Microspheres

The primary application of the microspheres synthesized in this study is as the stationary phase in preparative liquid chromatography columns. To evaluate the chromatographic performance of the silica microspheres, the monodisperse silica mesoporous microspheres with a narrow pore size distribution were utilized as the stationary phase to assemble a liquid chromatography column. Initially, 3 g of silica microspheres were dispersed in a mixture of 15 mL of cyclohexanol and 15 mL of acetonitrile using stirring and ultrasonication to create a homogenizer. Subsequently, the homogenizer was poured into a homogenizing tank, which was then connected to a column loader and filled with isopropanol as the displacing solvent. The column loader was activated, increasing the pressure from 3000 psi to 6500 psi, which was maintained until the displacement solvent, twice the volume of the homogenizer, was discharged. The pressure was then released, marking the completion of the column packing.

**Figure 11:**
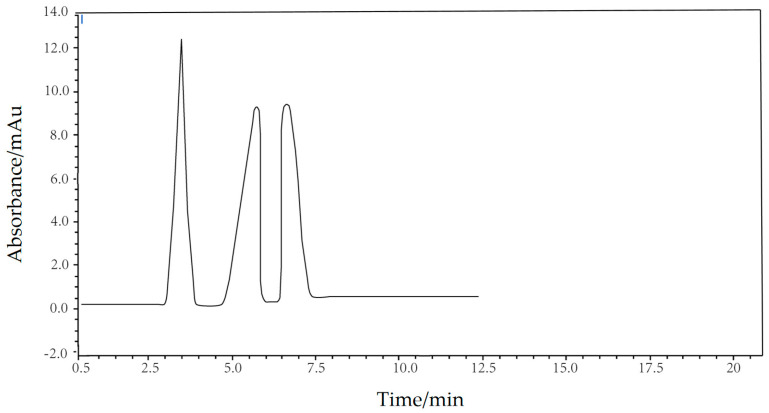
The chromatogram of alkylbenzene homologues separated using a column packed with silica mesoporous microspheres.

After assembling the chromatographic column with silica mesoporous microspheres as the stationary phase, it was installed into the liquid chromatograph. The mixture of toluene, 4–chloronitrobenzene, and nitrobenzene underwent chromatographic separation using normal phase chromatography. The chromatographic column’s separation efficiency for alkylbenzene homologues was evaluated, thereby assessing the chromatographic performance of the silica microspheres. Specific test parameters included a volume ratio of n–hexane to isopropanol in the mobile phase of 99.5:0.5, a detection wavelength of 254 nm, a column temperature of 25 °C, a flow rate of 1.0 mL/min, an injection volume of 10 μL, and concentrations of toluene, 4–chloronitrobenzene, and nitrobenzene in the normal phase analysis solution of 450, 15, and 20 μg/mL, respectively. The test results are depicted in Figure 11 and Table 7. It is observed from Figure 11 that the three peaks from left to right correspond to toluene, 4–chloronitrobenzene, and nitrobenzene. These peaks are distinctly separated, displaying sharp and complete profiles with a retention time of less than 10 min. This demonstrates that the chromatographic column with silica mesoporous microspheres as the stationary phase effectively separates alkylbenzene homologues. Table 7 presents the chromatographic data for the three alkylbenzene homologues, all with resolution factors exceeding 1.5, further confirming the superior separation efficiency of the column with silica mesoporous microspheres as the stationary phase.

## 4. Discussion

To prepare monodisperse mesoporous silica microspheres with narrow pore size distribution, we conducted an in–depth optimization of the synthesis formulation and process. The microspheres were then subjected to alkali–heating, calcination, and sieving. Additionally, we provided a detailed presentation and description of the experimental phenomena and results. Subsequently, we will interpret these experimental phenomena and results, delve into the formation mechanism of silica microspheres and their pore structures, evaluate the necessity and feasibility of the post–treatment process, and analyze the feasibility and associated issues of this preparation method for mass production.

Firstly, the experimental results indicate that only the silicon oil obtained at the appropriate temperature during rotary evaporation can form silica microspheres that are spherical, undamaged, and free of small particle agglomeration during the emulsification process. The silica sol undergoes a polymerization reaction during the rotary evaporation process, leading to the formation of silicon oil with a specific molecular weight and viscosity. The speed of the polymerization reaction is influenced by the temperature of rotary evaporation. As the temperature increases, the reaction speeds up, resulting in a higher degree of polymerization, molecular weight, and viscosity for the silicon oil. When the temperature of the rotary evaporation is too low, the degree of polymerization for the silicon oil is low, leaving hydroxyl groups on its surface, preventing the formation of stable spherical oil droplets through emulsification and ultimately resulting in an irregular, blocky structure. Conversely, when the temperature is too high, the viscosity of the silicon oil becomes too high, making it difficult to form spherical droplets due to insufficient shear forces generated by stirring. Instead, ellipsoidal droplets are formed, leading to an ellipsoidal structure. Under specific shear forces, the surface characteristics and viscosity of the silicon oil directly determine whether stable spherical droplets can be formed through an O/W emulsification process. The appropriate temperature of rotary evaporation ensures the hydrophobicity and appropriate viscosity of the silicon oil.

Furthermore, the experimental results indicate that variations in emulsification speed significantly affect the particle size of microspheres, while smaller adjustments in this speed have a more modest impact on the distribution of particle sizes. The speed of emulsification directly determines the level of shear force, such that higher speeds correspond to greater shear forces, which shear the oil droplets in O/W emulsions into smaller sizes. However, minor adjustments in the emulsification speed do not lead to significant changes in shear force, hence they have a minimal influence on particle size. Interestingly, this influence has been observed to affect the particle size distribution of microspheres. The microspheres prepared at 800 rpm exhibit better particle size distribution under SEM. We speculate that this observation is related to the stability of the emulsification equipment used, particularly at 800 rpm. This phenomenon offers valuable insights for the industrial production of silica microspheres, highlighting that the selection and adjustment of emulsification equipment can profoundly influence the quality of microspheres produced.

In the process of preparing silica mesoporous microspheres by the traditional emulsion method, surfactants are usually used to disperse the oil phase in the water phase, and templates are introduced to construct the mesoporous structure. However, the residual surfactants and templates may affect the purity of microspheres. To avoid the use of surfactants and templates, we chose to use the aprotic solvent DMF as a substitute. In the synthesis process, DMF plays the role of both dispersant and porogen. As a dispersant, when the dosage of DMF is small, the silicone oil cannot be evenly dispersed in the alcohol–water mixture, and the silicone oil agglomerates so that the final product appears as agglomerated block rather than dispersed microspheres, which is consistent with the experimental results. As a porogen, when the dosage of DMF is small, the pore size and pore volume of the microspheres are small. With the increase of DMF content, the median pore size of microspheres increased significantly, which was consistent with the experimental results. However, there is an abnormal phenomenon in the experimental results: excessive DMF leads to the decrease of pore volume, average pore size, and pore size distribution of the microspheres. We speculate that this may be because only when the appropriate proportion of DMF and silicone oil are miscible can a co–continuous miscible be formed. After that, the silicone oil gelled under the action of catalyst ammonia to form a continuous skeleton phase of silica microspheres. After the DMF was dried and removed, interpenetrating mesopores of silica microspheres were formed. When the dosage of DMF exceeds a certain threshold, the excess DMF will completely encapsulate the silicone oil in localized regions, leading to damage to the co–continuous structure and subsequently affecting the strength of the formed silica microspheres. This may cause some mesopores to collapse and shrink during subsequent drying, alkali–heating, and calcination.

In emulsion polymerization, NH_3_·H_2_O is used as a catalyst to accelerate the reaction process. During the stirring process, NH_3_·H_2_O diffuses into the droplets, causing the silicone oil in the droplets to gel, forming silica microspheres. The amount of NH_3_·H_2_O directly affects the speed and degree of gelation and then affects the strength of silica microspheres. When the amount of NH_3_·H_2_O is small, the skeleton strength of the silica microspheres is low, and the internal mesopores are more likely to shrink in the subsequent drying and calcination process. Therefore, the pore size and pore volume of the silica mesoporous microspheres are low, which are consistent with the experimental results. However, when the dosage of NH_3_·H_2_O is too much, the experimental results show that the pore volume of silica mesoporous microspheres decreases and the pore size distribution becomes wider. We speculate that this phenomenon may be related to the mechanism of NH_3_·H_2_O in the reaction system. When NH_3_·H_2_O is added to the emulsion, it will first be dispersed into the water phase. Subsequently, by stirring, NH_3_·H_2_O will gradually diffuse into the oil phase, thus promoting the gelation reaction and forming microspheres. This diffusion process requires a certain mass transfer time. When the dosage of NH_3_·H_2_O is too much, there will be a large NH_3_·H_2_O concentration gradient within the oil droplets, resulting in an uneven gelation velocity. This non–uniform gel structure will not only widen the pore size distribution of the microspheres, but also affect the subsequent pore expansion of alkali–heating, resulting in a decrease in pore volume.

Then, in view of the problems of small pore size and the wide particle size distribution of silica microspheres prepared only by emulsion method, we used alkali–heating and sieving to carry out post–treatment. In the process of alkali–heating, the alkali solution will erode the pores in the silica microspheres to achieve the purpose of pore expansion. However, when the concentration of alkali solution is too high, it will lead to a large concentration gradient of alkali solution in the mesoporous channel, which will make the pore expansion process become uneven, manifested in the broadening of pore size distribution from the experimental phenomenon. Therefore, we believe that it is very necessary to optimize the alkali–heating process. The principle of sieving is relatively simple. It mainly uses sieves with specific pore sizes to filter silica microspheres and remove those with sizes that are either too large or too small, thereby narrowing the particle size distribution of the microspheres. Although this method of sieving microspheres with sieves is relatively simple, easy to achieve in the laboratory, and effective, it cannot be used in mass production. There are three main reasons for this: (1) The efficiency of sieving with a sieve is low, making it difficult to meet the demands of mass production. (2) The mesh size accuracy required of the sieve in the sieving process is very high, and high–precision sieves are very expensive. (3) Sieves can easy become blocked by materials in the process of use and need to be cleaned frequently. Frequent cleaning will significantly affect the production efficiency and may damage the sieve. Therefore, researching and developing sieving methods more suitable for the mass production of silica microspheres has become an important research direction in this field.

Finally, we prepared a liquid chromatography column with silica mesoporous microspheres as the stationary phase, successfully separating three alkylbenzene homologues. Both the chromatogram and chromatographic data demonstrate the good chromatographic performance of the silica mesoporous microspheres developed in this study. Considering only the chromatographic performance, the silica mesoporous microspheres developed in this study show promising application potential in preparative liquid chromatography columns. However, in the practical application of microspheres as stationary phases, chromatographic performance represents only a minor aspect. The cost and stability of mass–produced microspheres largely determine their potential for commercial application in this field. Therefore, future research on the chromatographic performance and applications of the microspheres discussed in this paper should be founded on mass production, incorporating studies on the bonding of various functional groups to achieve effective application in diverse liquid chromatography modes.

## 5. Conclusions

In summary, in order to meet the requirements of preparative liquid chromatography columns, monodisperse silica microspheres with large particle size, large pore volume, and narrow pore size distribution were prepared by an improved emulsion method combined with alkali–heating, calcination, and a sieving process. The formation mechanism of silica microspheres and their pore structures were discussed in depth, and the necessity and feasibility of the alkali–heating and sieving processes were analyzed. The rotary evaporation temperature, emulsification speed, DMF dosage, NH_3_·H_2_O dosage, alkali–heating, and sieving processes have important roles in determining the morphology and pore structure of silica mesoporous microspheres. The silica mesoporous microspheres with a particle size (D_50_) of 26.3 μm, a D_90_/D_10_ ratio of 1.94, a pore volume of more than 1.0 cm^3^/g, an average pore size of 11.35 nm, and a median pore size of 13.4 nm can be achieved. The as–prepared silica mesoporous microspheres possess good chromatographic performance in the separation of alkylbenzene homologues. The silica mesoporous microspheres are promisingly applied in preparative liquid chromatographic columns.

## Figures and Tables

**Table 1:** Formula and process characteristics of all samples.

Sample	T ^a^/°C	R ^b^/rpm	V_DMF_ ^c^/mL	V_NH3·H2O_ ^d^/mL	Post–Treatment
1	50	600	9.6	16	/
2	53	600	9.6	16	/
3	57	600	9.6	16	/
4	60	600	9.6	16	/
5	65	600	9.6	16	/
6	60	700	9.6	16	/
7	60	800	9.6	16	/
8	60	1000	9.6	16	/
9	60	1200	9.6	16	/
10	60	8000	9.6	16	/
11	60	800	7.2	16	process A ^e^
12	60	800	9.6	16	process A ^e^
13	60	800	12.0	16	process A ^e^
14	60	800	14.4	16	process A ^e^
15	60	800	9.6	8	process A ^e^
16	60	800	9.6	12	process A ^e^
17	60	800	9.6	20	process A ^e^
18	60	800	9.6	16	process B ^f^
19	60	800	9.6	16	process C ^g^
20	60	800	9.6	16	process D ^h^

^a^ rotary evaporation temperature. ^b^ emulsification speed. ^c^ dosage of DMF. ^d^ dosage of NH_3_·H_2_O. ^e^ with alkali–heating of 0.14 mol/L NH_3_·H_2_O and calcination. ^f^ with calcination. ^g^ with alkali–heating of 0.07 mol/L NH_3_·H_2_O and calcination. ^h^ with alkali–heating of 0.21 mol/L NH_3_·H_2_O.

**Table 2:** Pore structure characteristics of the samples synthesized with various DMF contents.

V_DMF_ ^a^/mL	S_p_ ^b^/m^2^·g^−1^	V_p_ ^c^/cm^3^·g^−1^	D_a_ ^d^/nm	D_m_ ^e^/nm
7.20	271.0	0.507	7.250	/
9.60	365.3	1.020	11.35	13.4
12.0	330.1	0.894	10.16	18.3
14.4	331.3	0.854	9.680	18.7

^a^ dosage of DMF. ^b^ Brunauer–Emmett–Teller (BET) specific surface area. ^c^ Adsorption pore volume measured by Barrett–Joyner–Halenda (BJH) method. ^d^ Adsorption average pore diameter measured by BJH method. ^e^ Median pore diameter measured from unimodal BJH mesopore size distribution.

**Table 3:** Pore structure characteristics of the silica microspheres synthesized with various NH_3_·H_2_O contents.

V_NH3·H2O_ ^a^/mL	S_p_ ^b^/m^2^·g^−1^	V_p_ ^c^/cm^3^·g^−1^	D_a_ ^d^/nm	D_m_ ^e^/nm
8	306.8	0.450	5.797	9.20
12	314.7	0.510	6.425	9.40
16	365.3	1.020	11.35	13.4
20	314.0	0.744	9.503	17.0

^a^ dosage of NH_3_·H_2_O. ^b^ BET specific surface area. ^c^ Adsorption pore volume measured by BJH method. ^d^ Adsorption average pore diameter measured by BJH method. ^e^ Median pore diameter measured from unimodal BJH mesopore size distribution.

**Table 4:** Post–treatments for preparing the silica microsphere samples 1–5.

Sample	Post–Treatment (Alkali–Heating and Calcination)
1	Without alkali–heating and calcination
2	With calcination
3	With alkali–heating of 0.07 mol/L NH_3_·H_2_O and calcination
4	With alkali–heating of 0.14 mol/L NH_3_·H_2_O and calcination
5	With alkali–heating of 0.21 mol/L NH_3_·H_2_O and calcination

**Table 5:** Pore structure characteristics of the silica microsphere samples 1–5 with different post–treatments.

Sample	S_p_ ^a^/m^2^·g^−1^	V_p_ ^b^/cm^3^·g^−1^	D_a_ ^c^/nm	D_m_ ^d^/nm
1	753.3	0.898	3.979	4.82
2	696.7	0.735	3.751	4.26
3	381.8	0.979	10.17	12.1
4	365.3	1.020	11.35	13.4
5	325.8	0.960	11.94	18.2

^a^ BET specific surface area. ^b^ Adsorption pore volume measured by BJH method. ^c^ Adsorption average pore diameter measured by BJH method. ^d^ Median pore diameter measured from unimodal BJH mesopore size distribution.

**Table 6:** Particle size distribution data of the silica microspheres before and after sieving.

Sample	D_10_/μm	D_50_/μm	D_90_/μm	D_90_/D_10_
Before sieving	6.74	23.4	47.1	6.99
After sieving	18.1	26.3	35.2	1.94

**Table 7:** The chromatographic data of alkylbenzene homologues separated using a column packed with silica mesoporous microspheres.

Alkylbenzene Homologues	Retention Time/min	Peak Height/mAu	Plate Number	Resolution
toluene	3.54	12.493	794	2.12
4–chloronitrobenzene	5.92	9.352	872	1.78
nitrobenzene	6.65	9.675	855	1.53

## Data Availability

The data that support the findings of this study are available on request from the corresponding author upon reasonable request.
